# *LcMYB1* Is a Key Determinant of Differential Anthocyanin Accumulation among Genotypes, Tissues, Developmental Phases and ABA and Light Stimuli in *Litchi chinensis*

**DOI:** 10.1371/journal.pone.0086293

**Published:** 2014-01-21

**Authors:** Biao Lai, Xiao-Jing Li, Bing Hu, Yong-Hua Qin, Xu-Ming Huang, Hui-Cong Wang, Gui-Bing Hu

**Affiliations:** 1 State Key Laboratory for Conservation and Utilization of Subtropical Agro-Bioresources, College of Horticulture, South China Agricultural University, Guangzhou, Guangdong, People's Republic of China; 2 Physiological Laboratory for South China Fruits, College of Horticulture, South China Agricultural University, Guangzhou, People's Republic of China; Key Laboratory of Horticultural Plant Biology (MOE), China

## Abstract

The red coloration of litchi fruit depends on the accumulation of anthocyanins. The anthocyanins level in litchi fruit varies widely among cultivars, developmental stages and environmental stimuli. Previous studies on various plant species demonstrate that anthocyanin biosynthesis is controlled at the transcriptional level. Here, we describe a litchi R2R3-MYB transcription factor gene, *LcMYB1*, which demonstrates a similar sequence as other known anthocyanin regulators. The transcription levels of the *LcMYB1* and anthocyanin biosynthetic genes were investigated in samples with different anthocyanin levels. The expression of *LcMYB1* was strongly associated with tissue anthocyanin content. *LcMYB1* transcripts were only detected in anthocyanin-accumulating tissues and were positively correlated with anthocyanin accumulation in the pericarps of 12 genotypes. ABA and sunlight exposure promoted, whereas CPPU and bagging inhibited the expression of *LcMYB1* and anthocyanin accumulation in the pericarp. *Cis-*elements associated with light responsiveness and abscisic acid responsiveness were identified in the promoter region of *LcMYB1*. Among the 6 structural genes tested, only *LcUFGT* was highly correlated with *LcMYB1*. These results suggest that *LcMYB1* controls anthocyanin biosynthesis in litchi and *LcUFGT* might be the structural gene that is targeted and regulated by *LcMYB1*. Furthermore, the overexpression of *LcMYB1* induced anthocyanin accumulation in all tissues in tobacco, confirming the function of *LcMYB1* in the regulation of anthocyanin biosynthesis. The upregulation of *NtAn1b* in response to *LcMYB1* overexpression seems to be essential for anthocyanin accumulation in the leaf and pedicel. In the reproductive tissues of transgenic tobacco, however, increased anthocyanin accumulation is independent of tobacco's endogenous MYB and bHLH transcriptional factors, but associated with the upregulation of specific structural genes.

## Introduction

The colors of flowers and fruits mainly result from the accumulation of anthocyanins, a group of secondary metabolites that are synthesized via the flavonoid pathway [1]. The structural genes involved in the anthocyanin biosynthetic pathway of plants include chalcone synthase (CHS), chalcone isomerase (CHI), flavanone 3-hydroxylase (F3H), flavonoid 3′-hydroxylase (F3′H), dihydroflavonol 4-reductase (DFR), anthocyanidin synthase (ANS), and UDP-glucose: flavonoid 3-*O*-glucosyltransferase (UFGT). These genes are well characterized in model plants [2], [3] and fruits including grape [4], apple [5], Chinese bayberry [6], and litchi [7]. In addition, numerous studies demonstrate that anthocyanin accumulation is largely regulated at the transcriptional factors which manipulate the expression of structural genes in the anthocyanin biosynthetic pathway [8].

Among various complex transcription factors [including MYB, basic helix-loop-helix (bHLH) and WD40 protein], MYB appears to be the major determinant of anthocyanin accumulation [9], [10]. Subgroup 6 clade MYBs, *PAP1* and *PAP2*, in *Arabidopsis* and their orthologs in other plant species are known to be the key regulators of anthocyanin biosynthesis. *PAP1* and *PAP2* overexpression in *Arabidopsis* induces anthocyanin biosynthesis in the entire plant [11], [12]. Many MYBs have been identified to control the accumulation of anthocyanin in the tissue or organ of various plants, such as *GmMYB-G20-1* in soybean flower [13], [14], *BoMYB2* in purple cauliflower [15], *LhMYB6* and *LhMYB12* in Asiatic hybrid lily [16], *VvMYBA* in grape [17], [18], and *MrMYB1* in bayberry [19]. In apple, the red color of the fruit skin and flesh are controlled by two MYB genes, namely *MdMYBA* and *MdMYB10* [20], [21]. Some MYB transcription factors, however, inhibit the accumulation of anthocyanin. Two R3-MYBs were reported to negatively regulate anthocyanin in *Arabidopsis* [22], [23]. In strawberry, *FaMYB1* represses transcription of anthocyanin-related genes during maturation, and the biosynthesis of proanthocyanidins is inhibited in the leaves of *FaMYB1* transgenic *Lotus corniculatus* [24], [25]. These results suggest functional differences between various R2R3-MYB proteins.

Expression pattern analysis and DNA-protein interaction assays indicate that different MYBs activate different anthocyanin biosynthetic structural genes. *PAL*, *CHS*, and *DFR* are activated in *pap1*-*D* plants by linking an enhancer sequence to *PAP* [11]. In grape berry, *MybA* gene regulates anthocyanin biosynthesis via *UFGT* expression [17]. *MdMYB1* cDNA increases luciferase enzyme activity when cobombarded with constructs containing the *MdDFR* and *MdUFGT* promoters [26]. This suggests that *MdMYB1* can activate the expression of these two anthocyanin pathway genes in apple. Similarly, the activity of *Arabidopsis* DFR promoter was significantly increased when *MdMYB10* was cotransformed with an apple bHLH [21]. The coexpression of *GbMYB2* and *GbMYC1* activates the *GbDFR* and *GbANS* promoters in tobacco leaf protoplasts [27]. *Pr-D* overexpression in cauliflower specifically activates a bHLH transcription factor and a subset of anthocyanin structural genes that encode *BoF3′H*, *BoDFR*, and *BoANS* [28].

It is well known that both intrinsic and extrinsic factors affect the biosynthesis of anthocyanins. Anthocyanin accumulation is influenced by various environment conditions, such as light, temperature, and phytohormones. In apple [26], pear [29], peach [30], and bayberry [6], anthocyanin biosynthesis is enhanced by sunlight. Generally, shading fruit by bagging with a dark material inhibits the biosynthesis of anthocyanins, and rapid anthocyanin accumulation is noticeable after bag removal. High temperature inhibits, while low temperature enhances anthocyanin accumulation in apple fruit [31], [32]. ABA promotes anthocyanin synthesis during grape development and grape cell culture [33]–[35]. Jasmonate (JA) regulates anthocyanin accumulation in *Arabidopsis* by degrading JA ZIM-domain (JAZ) proteins, preventing the interactions between JAZ proteins and bHLH and MYB transcription factors [36].

Litchi (*Litchi chinensis* Sonn.) is an economically important evergreen fruit crop in the Sapindaceae family. The fruit consist of drupes with a white translucent edible aril that is surrounded by the pericarp. The red color on the pericarp of litchi results from the accumulation of anthocyanins [37]. Color differences between litchis are mainly due to variations in the anthocyanin concentration in the pericarp. The biosynthesis of anthocyanins in the pericarp of litchi demonstrates cultivar, developmental, and environmental differences. And one structural gene, *LcUFGT*, has been shown to have major role in these differences [7]. However, this mechanism has not been characterized at the level of transcriptional regulation.

In this study, an R2R3-MYB gene, *LcMYB1*, was isolated from the pericarp of ‘NMC’, a red litchi cultivar. Its expression patterns in different tissues, pericarp developmental stages, color genotypes, and ABA and light stimuli were assessed, and the correlation between anthocyanin accumulation and the expression of other anthocyanin biosynthetic genes was investigated. Furthermore, the functional activity of this transcription factor in driving anthocyanin accumulation was evaluated using two heterologous transgenic assays. In these studies, the behavior of *LcMYB1* suggests that it is responsible for red pigmentation in litchi fruit. ABA and sunlight exposure enhance anthocyanin accumulation, mainly by upregulating *LcMYB1* expression. The overexpression of *LcMYB1* in transformed tobacco lines indicates that the efficient induction of anthocyanin biosynthesis by *LcMYB1* in vegetative tissues depends on the coexpression of the bHLH protein, *NtAn1b*.

## Results

### Isolation of *LcMYB*s from the pericarp of litchi

**Figure 1:**
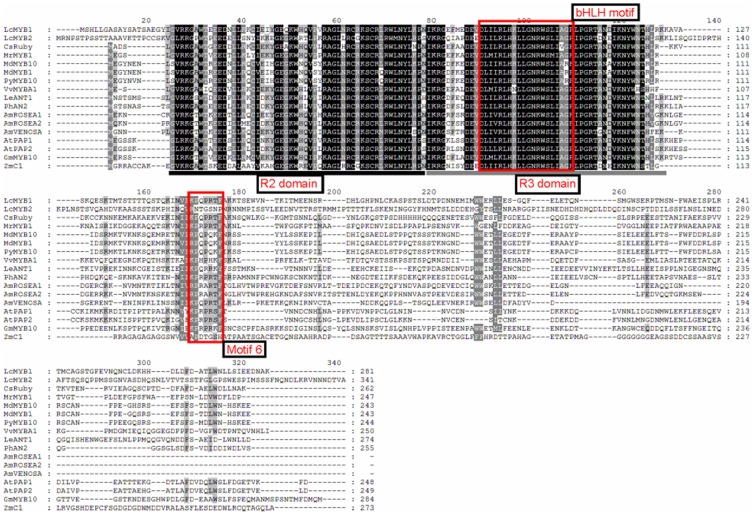
Protein sequence alignment of the R2R3 DNA-binding domains of *LcMYB1* and the other known anthocyanin MYB regulators in other species. The R2 and R3 domains are underlined. The bHLH-binding motif is boxed in the R3 domain. Motif 6 was previously identified in the C-terminal domain of anthocyanin-related MYBs in *Arabidopsis* [39]. The accession number of these proteins (or translated products) are as follows in the GenBank database: *MrMYB1*, GQ340767; *MdMYB1*, ABK58136; *MdMYB10*, DQ267896; *PyMYB10*, ADN26574; *GmMYB10*, ACM62751.1; *PhAN2*, AAF66727; *LeANT1*, AAQ55181; *CsRuby*, AFB73909; *MrMYB1*, GQ340767; *VlMYBA1*-1, BAC07537; *VvMYBA1*, BAD18977; *VvMYBA2*, BAD18978; *GhMYB10*, CAD87010; *AtPAP1*, AAG42001; *AtPAP2*, AAG42002; *AtMYB113*, NP_176811; *AtMYB114*, NP_176812; *AmROSEA1*, ABB83826; *AmROSEA2*, DQ275530; *AmVENOSA*, DQ275531; and *FaMYB1*, AF401220.

Using degenerate primers, a 283-base pair (bp) product was amplified using ‘NMC’ cDNA. The full-length cDNA was obtained using 3′ and 5′ RACE. This full-length cDNA, namely *LcMYB1*, is a 940 bp transcript that encodes a protein of 281 amino acids. When translated into protein, *LcMYB1* displays distinct R2 and R3 MYB repeat domains (Fig. 1). In the R3 domain, *LcMYB1* shows residues that in accordance with amino acid motif [DE]Lx2[RK]x3Lx6Lx3R predicted to allow interaction with bHLHs, suggesting that it may interact with bHLH proteins [38]. In the variable region, the small motif [K/R]Px3[K/T][F/Y] is conserved [26]. This motif is part of the motif that was previously reported as KPRPR[S/F]F (motif 6) [39]. *LcMYB1* demonstrated a high degree of homology with other MYB transcription factors (Fig. 1). The amino acid identity over the R2R3 DNA-binding domain are 86.7% similar to Chinese bayberry *MrMYB1*, 83.8% similar to *Arabidopsis PAP1*, 81.9% similar to grape *VvMYBA1*, and 80.0% similar to apple *MdMYB10* respectively.

**Figure 2:**
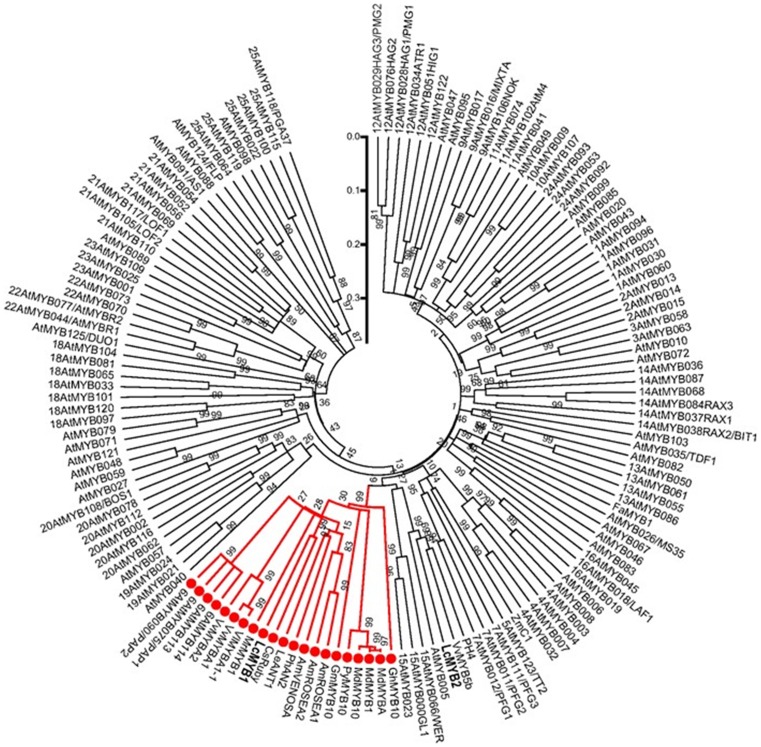
Phylogenetic relationships between *Arabidopsis* MYB transcription factors and anthocyanin-related MYBs in other species. *LcMYB1* clusters next to *CsRudy*, within the anthocyanin subgroup 6 MYBs. The subgroup numbers were previously described [12]. The tree was constructed using MEGA 5, neighboring-joining phylogeny testing, and 1,000 bootstrap replicates. The accession numbers for the genes in the other species are provided in Figure 1.

The phylogenetic analysis indicates that *LcMYB1* is closely related to the subgroup 6 MYBs in *Arabidopsis*: the nearest MYB is *CsRuby*, which shared 87.1% and 49.3% amino acid identity with the R2R3 DNA-binding domain and the entire protein, respectively (Fig. 2). *LcMYB1* is clustered with the R2R3 MYB transcription factors that are involved in the regulation of anthocyanin biosynthesis in other plant species. These results suggest that *LcMYB1* may demonstrate similar functions as subgroup 6 MYBs in *Arabidopsis* [12], which play an important role in regulating anthocyanin synthesis.

In addition, *LcMYB2*, which is homologous to *LcMYB1*, was isolated from our transcriptome sequences data, and full-length cDNA was obtained using 3′and 5′ RACE. However, the expression pattern and phylogenetic analysis demonstrated that *LcMYB2* may not be involved in the regulation of anthocyanin biosynthesis (Fig. S1); thus, *LcMYB2* was not further analyzed in this paper.

### Anthocyanin contents and the expressions of biosynthetic genes in litchi

**Figure 3:**
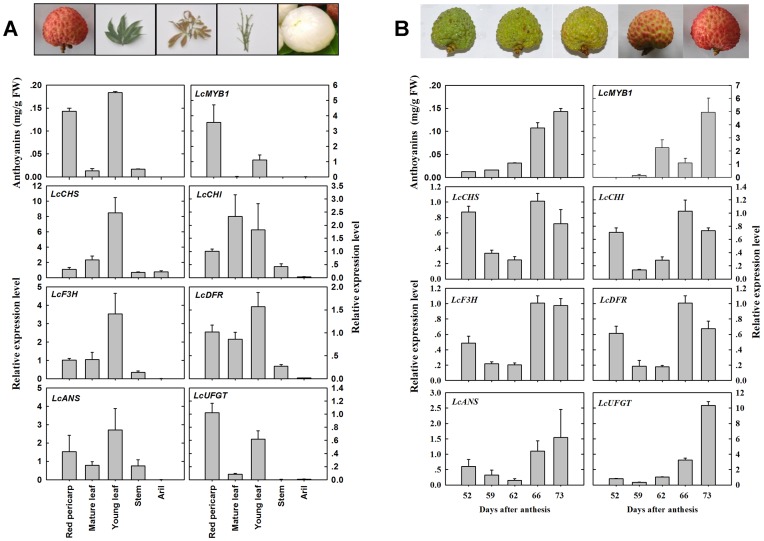
Anthocyanin contents and expressions of biosynthetic genes. (A) Anthocyanin contents and expression analysis of *LcMYB1* and anthocyanin biosynthetic structural genes in different tissues of ‘NMC’ litchi including red pericarp, mature and young leaves, young stem and aril. (B) Anthocyanin contents and expression analysis of *LcMYB1* and anthocyanin biosynthetic structural genes in the pericarp of ‘NMC’ litchi during fruit development. The *Lcactin* gene was used to normalize the expression levels of the genes under identical conditions. The vertical bars represent the standard error of triplicate experiments.

The total anthocyanin content in different tissues of ‘NMC’ was measured using the pH-differential spectrum method. Content varies greatly between different tissues (Fig. 3 A). Red tissues, including mature pericarp and young leaf, contained much higher levels of anthocyanins, while extremely low or no anthocyanins was detected in non-red tissues, including mature leaf, young stem, and aril.

The transcription levels of *LcMYB1* and the structural genes that encode the enzymes of the anthocyanin pathway were determined in different litchi tissues using qRT-PCR and gene-specific primers (Fig. 3 A). Notable differences were observed. Extremely low or undetectable levels of *LcMYB1* and the six tested structural genes were noticed in non-red stem and aril. In non-red mature leaf, the expression levels of *LcMYB1* and *LcUFGT* were also low or undetectable, while the expression levels of other genes including *LcCHS*, *LcCHI*, *LcF3H*, *LcDFR* and *LcANS* were detectable. In red pericarp, relatively high transcription levels of *LcMYB1*, *LcDFR*, *LcANS*, and *LcUFGT* were observed. The expression levels of *LcMYB1* and the six structural genes were all high in the anthocynin-rich young leaf.

The developmental patterns of anthocyanin content and the expression levels of *LcMYB1* and the six structural genes in the pericarp of ‘NMC’ are shown in Figure 3 B. The rapid accumulation of anthocyanins occurred during the late stage of fruit development. The content of anthocyanins was low (<0.03 mg g^−1^ FW) in the pericarp before 62 days after full bloom (DAFB), but increase dramatically to 0.11 mg g^−1^ FW and 0.14 mg g^−1^ FW at 66 and 73 DAFB, respectively. In parallel with the accumulation of anthocyanins, the expression levels of *LcMYB1* and *LcUFGT* were enhanced as the fruit developed toward full maturity. For the rest of the structural genes, the expression levels were relative high at the first sampling date, decreased afterwards, and increased along with rapid anthocyanin accumulation.

### Cultivar differences and the effects of manipulation treatments on *LcMYB1* expression

**Figure 4:**
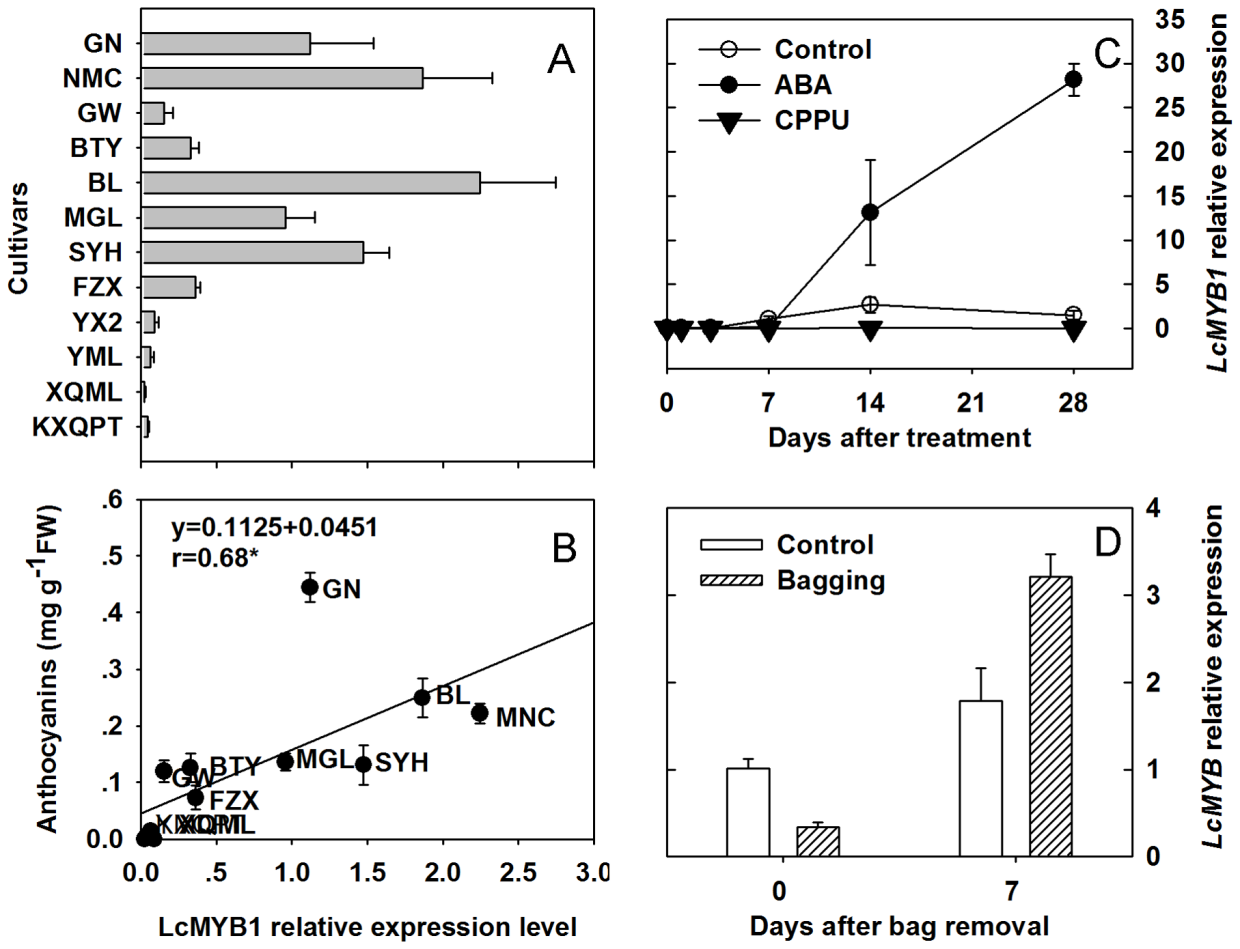
*LcMYB1* expression in the pericarp of different cultivars and manipulation treatments. A, *LcMYB1* expression of in the pericarp of twelve cultivars. B, The correlationship between *LcMYB1* expression and anthocyanin content among the twelve cultivars. Anthocyanin contents were obtained from Wei et al. [7], Effects of ABA and CPPU on the expression of *LcMYB1*. D, Effects of bagging and bag removal on *LcMYB1* expression.

According to our previous study [7], anthocyanin content in the pericarp varies greatly between the 12 tested cultivars. In the present study, the expression of *LcMYB1* and its correlation with anthocyanin in the pericarps of these 12 cultivars was assessed. Consistent with pericarp anthocyanin content, the non-red cultivars ‘KXQPT’, ‘XQML’, ‘YML’ and ‘YX2’ displayed extremely low levels of *LcMYB1* expression, while noticeable *LcMYB1* expression was observed in the rest red cultivars (Fig. 4 A). The expression of *LcMYB1* was significantly and positively correlated with anthocyanin content in the pericarps of the tested cultivars (r = 0.68) (Fig. 4 B).

The expression levels of *LcMYB1* in response to the ABA and CPPU treatments are shown in Figure 4 C. ABA enhanced while CPPU inhibited anthocyanin accumulation in ‘Feizixiao’ [7]. The expression patterns of *LcMYB1* were consistent with anthocyanin accumulation across different treatments. *LcMYB1 e*xpression was detected in all of the red pericarps, but not in any of the non-red pericarps. Expression was undetectable before 14 days after treatment (DAT), after which there was a notable expression in the control. In CPPU-treated pericarp, the expression of *LcMYB1*was hardly detectable throughout the entire experiment period, while a steep increase in *LcMYB1* expression was observed 7 days after ABA application.

Bagging and bag removal were used to study the effects of illumination on *LcMYB1* expression (Fig. 4 D). In the pericarp of the control fruit, which were grown under normal light conditions, *LcMYB1* transcription was approximately 3-fold higher than in the bagged fruit. When the dark-grown litchi fruit were re-exposed to sunlight, *LcMYB1* transcription increased by approximately 2-fold within 7 days after bag removal. The effects of bagging and bag removal on the expressions of *LcMYB1* in the pericarp generally paralleled the effects on anthocyanin accumulation [7]. This result is similar to the *MdMYB1* transcription patterns observed in response to illumination [26].

### *LcMYB1* function in tobacco

**Figure 5:**
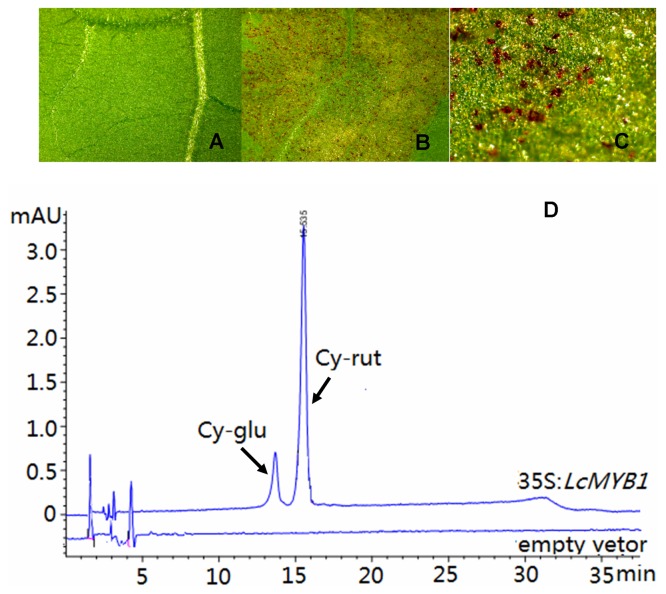
Color development in *Nicotiana tabacum* leaves following transient transformation. Microscopic images showing anthocyanin accumulation in tobacco leaf infiltrated with: A) an empty vector (1× magnification) or B–C) 35S:LcMYB1 (8× magnification). D) Anthocyanin HPLC profiles of 35S:LcMYB1 extracts from tobacco leaf (top line) and empty vector (bottom line). Peaks identified at 520 nm: cy-glu, cyanidin-3-glucoside; cy-rut, cyanidin-3-rutinoside.

The transient expression levels of anthocyanin-synthesizing transcription factors to prove their function in tobacco leaf are frequently utilized [6], [21], [40]. Pigmentation is evident at the infiltration points on tobacco leaf at 5 days after infiltration with *LcMYB1* cDNA (Fig. 5 A–C). To confirm that anthocyanins were synthesized in tobacco after *LcMYB1* transformation, leaf tissue sample were extracted and the anthocyanins were analyzed using HPLC. No observable anthocyanin peaks were observed in the extracts of the tobacco leaves that were transformed with the *Agrobacterium-* carrying empty vector, while two major peaks were observed in the extracts of the tobacco leaves that transformed with the *Agrobacterium-*carrying 35S:*LcMYB1* vector (Fig. 5 D). These two peaks represent cyanidin-3-glucoside and cyanidin-3-rutinoside respectively, which is consistent with the anthocyanin composition observed in the red pericarp of litchi.

**Figure 6:**
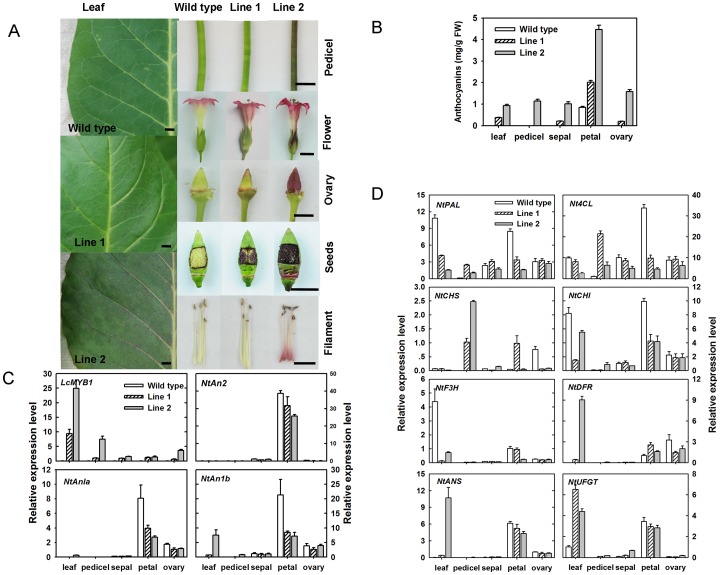
Images of the transgenic tobacco lines and the anthocyanin content, and the expression of anthocyanin biosynthetic pathway structural and regulatory genes in transgenic tobacco lines. A, Images of tobacco lines containing the empty vector (wild type) or the *LcMYB1* allele with the CaMV 35S promoter (Line 1–2) (Scale bars for images = 1cm. B, Anthocyanin content in different organs of transgenic tobacco lines. C, Regulatory gene expression in the anthocyanin biosynthetic pathway in transgenic tobacco. D, Structural gene expression in the anthocyanin biosynthetic pathway in transgenic tobacco lines. The *Lcactin* gene was used to normalize gene expression of the genes under identical conditions. The vertical bars represent the standard error of triplicate experiments.

The *LcMYB1* cDNA was fused with the CaMV 35S promoter and cloned into the binary pBI121 vector. To assess *LcMYB1* function, *Nicotiana tabacum* cultivar K326 leaf strips were transformed with 35S:*LcMYB1* and an *Agrobacterium-*mediated kanamycin selectable marker. Pigmented kanamycin-positive regenerated plantlets were noticed (Fig. S2). Pigmented leaf, pedicel, ovary, seed, filament, and highly pigmented petals were observed in the grown transformant lines, while the wide type plants demonstrated no obviously pigmented tissues except the petals (Fig. 6 A). The color of transformant line 2 was more intense than line 1. *LcMYB1* overexpression in tobacco resulted in anthocyanin accumulation in many tissues. Anthocyanins were detected in the leaves, pedicels, sepals, and ovaries of the transformant lines, especially line 2 (Fig. 6 B). Anthocyanin contents in the petals of the transformant line 1 and line 2 were 2.01±0.09 and 4.46±0.20 mg g^−1^ FW, respectively, which were much higher than that in the wild type (0.84±0.05 mg g^−1^ FW).

Furthermore, the expression levels of four regulatory factors were investigated, including *LcMYB1*, three tobacco anthocyanin biosynthesis regulators, and eight structural biosynthetic genes (Fig. 6 C–D). Distinct *LcMYB1* expression was observed in all of the tested tissues of the transformant lines, but no expression was detected in the tissues of wild type (Fig. 6 C). This result confirms the successful transformation of *LcMYB1*. Higher *LcMYB1* expression levels were observed in the leaves in comparison with the other tissues, and higher expression levels were observed in line 2. *NtAn2*, a R2R3-MYB transcription factor, interacts with bHLH regulator *NtAn1a* and *NtAn1b* to regulate the anthocyanin biosynthesis in tobacco flowers [41], [42]. In the present study, these endogenous regulatory factors (*NtAn2*, *NtAn1a* and *NtAn1b*) were specifically expressed in tobacco petals with extremely low or undetectable expression levels in other tissues (Fig. 6 C). The expression of *NtAn2* in petals decreased in response to increased *LcMYB1* expression. *NtAn1a* and *NtAn1b* were also downregulated in the petals of *LcMYB1*-overexpressing lines. In the leaf and pedicel, however, *NtAn1b* was upregulated especially in line 2.

In leaves, the expression levels of early and mid anthocyanin biosynthetic structural genes including *NtPAL*, *Nt4CL*, *NtCHS*, *NtCHI* and *NtF3H*, were all downregulated, whereas the expression levels of late anthocyanin biosynthetic genes, including *NtDFR*, *NtANS*, and *NtUFGT*, were all upregulated in transformant lines in comparison with wild type (Fig. 6 D). The expression levels of all structural genes in pedicel of wild type were extremely low, while upregulation of *NtPAL*, *Nt4CL*, and *NtCHS* was observed in the pedicels of the transformant lines. No obvious expression patterns, except for *NtUFGT*, were noticed in the sepals and ovaries of *LcMYB1-*overexpressing lines and wild type. In agreement with the anthocyanin content, the expression of *NtUFGT* was higher in the sepals and ovaries of the transformant lines, especially line 2, in comparison with wild type. Although the anthocyanin content was much higher in the petals of the transformant lines, the downregulation of most structural genes was noticeable. *NtDFR* was the only upregulated structural gene observed in both transformant lines.

### *LcMYB1* promoter analysis

Anthocyanin content and MYB transcription can be affected by exogenous stimulations, such as light, temperature, and hormones. To investigate the elements that could be associated with these factors, we isolated and analyzed the promoter region of *LcMYB1*. A 1.5-kb region upstream from the start codon of the *LcMYB1* promoter was predicted in PlantCARE database. The putative *cis-*elements identified in the *LcMYB1* promoter are showed in Table 1. The promoter sequence displayed multiple light-regulating units. ACE, Box 4, G-Box and GA-motif are involved in light responsiveness. Two ABRE are involved in the abscisic acid responsiveness. The remaining c*is*-elements include two HSE elements involved in the heat stress response, CGTCA and TGACG motifs involved in MeJA-responsiveness, a gibberellin-responsive element (GARE) motif, and an auxin-responsive (TGA) element. In addition, two binding motifs for MYB proteins were also found in the upstream region.

**Table 1:** Putative c*is*-elements identified in the *LcMYB1* promoter.

Category	*cis*-acting element	Sequence	Distance from ATG
Light response	ACE	AAAACGTTTA	+716
	Box 4	ATTAAT	−124, +585, +610
	G-Box	CACGTT	+475, +714, +1440
	G-Box	GACACGTAGT	−477
	G-Box	CACGTC	+135
	G-Box	TAAACGTG	−714
	G-Box	CACGTGG	−476
	GA-motif	ATAGATAA	+652, +1221
	GAG-motif	AGAGAGT	−1475
Abscisic acid response	ABRE	GGACACGTGGC	−477
	ABRE	CACGTG	+475
MeJA response	CGTCA-motif	CGTCA	−112
	TGACG-motif	TGACG	+112
Gibberellin response	GARE-motif	TCTGTTG	+208
	TATC-box	TATCCCA	−1356
Auxin-response	TGA-element	AACGAC	−324
MYBHv1-binding site	CCAAT-box	CAACGG	+427
MYB-binding site involved in drought-inducibility	MBS	TAACTG	−1250
Heat stress response	HSE	AAAAAATTTC	+493, +1071
Defense and stress	TC-rich repeats	ATTTTCTTCA	−1246

+, distance from ATG in a positive DNA strand; −, distance from ATG in a negative DNA strand.

## Discussion

### *LcMYB1* is homologous with other R2R3-MYBs involved in regulating anthocyanin biosynthesis

The pericarps of litchi demonstrate variations in anthocyanin accumulation depending on the cultivar, developmental stage, and environmental stimuli. It is possible that non-red pericarp lost the ability to produce anthocyanin due to a mutation in one or more biosynthetic or regulatory gene(s). As observed in white grapes [43], late biosynthetic genes, such as *LcDFR* and *LcUFGT* in particular, are coordinately expressed during the red coloration of litchi fruits [7]. It is well established that MYB transcriptional factors control the accumulation of anthocyanin in model plants [11], [44], [45]. In recent years, numerous MYBs responsible for anthocyanin biosynthesis in different kinds of fruits have been identified and characterized [6], [17], [20], [21], [24], [26], [29]. In the present study, the 940-bp full-length *LcMYB1* gene was isolated from the red pericarp of ‘NMC’. Sequence analysis of the *LcMYB1*-encoded protein confirmed its homology with anthocyanin-regulating MYB transcription factors in other species (Fig. 1). The *LcMYB1*-encoded protein exhibited distinct R2 and R3 MYB repeat domains and one signature motif which was predicted to interacted with bHLH. *LcMYB1* was clustered within the anthocyanin subgroup 6 MYBs and was highly orthologous with *CsRuby*, *MrMYB1*, *MdMYB10*, and *MdMYB1* (Fig. 2). These results suggest that *LcMYB1* maybe a member of the MYB anthocyanin activators.

### Key role of *LcMYB1* in anthocyanin accumulation

Most MYBs demonstrate tissue-specific expression characteristics. In apple, *MdMYB10* can be detected only in the leaf, cortex, and skin where containing large amount of anthocyanins [21], [40]. Similarly *LcMYB1* transcripts can be detected only in anthocyanin-rich tissues in litchi, i.e., red pericarp and young leaf (Fig. 3 A). In addition, the transcription level of *LcMYB1* is undetectable or extremely low in non-red pericarp, but is upregulated during fruit development and pigmentation (Fig. 3 B). The *LcMYB1* expression patterns in different tissues and fruit development stages are consistent with *MrMYB1* in bayberry and *VvMYBA* in wine grape [6], [17]. The expression levels of *LcMYB1* were weak in non-red cultivars (‘KXQPT’, ‘XQML’, ‘YML’ and ‘YX2’), but notable in red cultivars (‘FZX’, ‘SYH’, ‘MGL’, ‘BL’, ‘GW’, ‘NMC’ and ‘GN’) (Fig. 4 A). In sweet potato, *IbMYB1* is also expressed in purple-fleshed cultivars, but not in those of orange-, yellow-, or white-fleshed cultivars [46]. *LcMYB1* expression was positively correlated with anthocyanin accumulation in the pericarp of the 12 litchi cultivars (Fig. 4 B).

In non-climacteric strawberry, ABA is a signal molecule that promotes fruit ripening including the accumulation of anthocyanin [47]. However, the mechanisms in which ABA enhance anthocyanin synthesis in fruit have not yet been established. In grape berry, exogenous ABA rapidly induces the accumulation of anthocyanin and the expression of the corresponding structural genes [48]. Litchi is a typical non-climacteric fruit, and the endogenous ABA concentrations in the pericarp increases along with the synthesis of anthocyanins [49]. ABA significantly enhances anthocyanin accumulation in pericarp (while cytokinins inhibits accumulation), and decrease in endogenous ABA caused by cytokinins has also been observed [49]. Applying ABA 1 month before commercial harvest enhanced (whereas CPPU inhibited) *LcUFGT* expression and anthocyanin synthesis in the pericarp of litchi [7]. In the present study, ABA promoted, while CPPU reduced, the expression of *LcMYB1* (Fig. 4 C). It is possible that ABA affects the upstream gene *LcMYB1*, which regulates *LcUFGT* expression and subsequently the accumulation of anthocyanin in the pericarp of litchi. CPPU inhibits anthocyanin accumulation probably by decreasing endogenous ABA. Putative ABA responsive elements (ABRE) are identified in the promoter region of *LcMYB1* (Table 1). The mechanism that ABA enhances MYB expression and anthocyanin accumulation requires further study.

Bagging experiments have revealed that sunlight is essential for anthocyanin synthesis in apple [26], pear [19], peach [30], bayberry [6] and litchi [7]. Sunlight affects both anthocyanin accumulation and the expression of both anthocyanin biosynthetic genes and/or regulatory genes in these fruits. In the present study, *LcMYB1* expression is reduced in the pericarp of bagged fruit, but markedly upregulates when re-exposed to sunlight (Fig. 4 D). It is likely that sunlight regulates anthocyanin synthesis via *LcMYB1* expression. Constitutive Photomorphogenic 1 (COP1) is a RING-finger type ubiquitin E3 ligase involved in proteolysis during light signaling [50]. Recent studies have shown that COP1 can influence the stability of MYB transcription factors in the dark, thereby reducing anthocyanin accumulation in *Arabidopsis* and apple [51], [52]. Numerous putative light responsive elements have also been identified in the promoter region of *LcMYB1* (Table 1).

We use both transient transformation in tobacco leaves and stable tobacco transformants to assess the function of *LcMYB1* in the anthocyanin synthesis pathway. Microscopic images and HPLC data show the significant accumulation of anthocyanin compounds in transient expression tobacco leaves, confirming the functional presence of anthocyanin biosynthetic enzymes in tobacco leaves and the presence of the cellular machinery needed to transport anthocyanins to the vacuole (Fig. 5). The transient expression of *LcMYB1* in tobacco results in two anthocyanin peaks, representing cyanidin-3-glucoside and cyanidin-3-rutinoside, which is consistent with the peaks identified in the pericarps of red litchi cultivars [7].

When *LcMYB1* was stably transformed in tobacco, anthocyanin accumulation is noticed not only in the reproductive organ but also in the leaf and pedicel (Fig. 6 A). Considerable amounts of anthocyanins were detected in the leaves, pedicels, sepals, petals and ovaries of transgenic lines, especially line 2 (Fig. 6 B). These results suggested that *LcMYB1* is responsible for controlling anthocyanin biosynthesis in litchi.

### Coordinately regulation of litchi anthocyanin biosynthesis

Retrotransposon-induced insertion into the grape anthocyanin regulatory gene *VvmybA1* resulted in a lack of anthocyanin biosynthesis and the development of white grape cultivars [18]. Sicilian blood orange arose by insertion of a Copia-like retrotransposon adjacent to a gene encoding Ruby, a MYB transcriptional activator of anthocyanin production [53].

MYBs control the biosynthesis of anthocyanins by activating the expression of structural genes [8]. Structural genes and MYBs are usually coordinately expressed during anthocyanin synthesis. In Chinese bayberry, the relative intensities of *MrF3H*, *MrF3*′*H*, *MrDFR1*, *MrUFGT* and *MrMYB1* mRNA are strongly associated with anthocyanin content [6]. In apple, the expression of *MdANS*, *MdUFGT*, and *MdMYB1* demonstrate a positive correlation with anthocyanin synthesis in cultivars that show different colors [26]. Our previous study on different fruit color genotypes of litchi demonstrates that late genes in the anthocyanin biosynthetic pathway, *LcDFR* and *LcUFGT* in particular, are coordinately expressed during red coloration [7]. In the present study, *LcUFGT* expression was highly correlated with *LcMYB1* expression in different tissues and different fruit developmental stages in litchi (Fig. 3 A–B). The other genes were less influenced by tissue and fruit developmental stage. Relatively high expression levels of these genes were observed in non-red mature leaf and green pericarp. This result is consistent with studies on grapes that report *UFGT* as the only gene that makes the difference in coloration between white type and its red sport [42], [54].

### Response of tobacco endogenous genes in anthocyanin biosynthesis to *LcMYB1* overexpression

In apple and Chinese bayberry, without co-infiltrate with a bHLH transcription factor, the functions of *MdMYB10*, *MdMYB110a* and *MrMYB1* were quite weak in transient expression tobacco leaf [6], [21]. In the present study, *LcMYB1* alone effectively induced anthocyanin accumulation in tobacco leaf (Fig. 5). Interestingly, pigmented organs varied between tobacco lines that were transformed with MYBs from different species. When *PAP1* or *NtAn2* are overexpressed in tobacco, the whole plant produces anthocyanins [41], [55]. However, only petals and seeds accumulate anthocyanins in *MdMYB1* and *MrMYB1* transformed tobacco [19], [20]. This difference might result from the abilities of the exogenous MYBs and the interactions with endogenous bHLH. When *CsRuby* alone is overexpressed in tobacco, no anthocyanins are observed; when combined with different bHLH transcription factors, tobacco leaves accumulate different concentrations of anthocyanins [53].

The whole plant of *LcMYB1* overexpression line 2 produces anthocyanins (Fig. 6 A–B). The responses of endogenous MYB and bHLH transcription factors and anthocyanin biosynthetic genes to the overexpression of *LcMYB1* were investigated in tobacco in the present study (Fig. 6 C–D). Different tissues responded differently to *LcMYB1* overexpression in terms of endogenous gene expression. Higher expression levels of endogenous leaf *NtAn1b* and late anthocyanin biosynthetic genes, including *NtDFR*, *NtANS*, and *NtUFGT*, were observed in both transgenic tobacco lines. In the pigmented pedicel of line 2, the upregulation of *NtAn1b* and *NtUFGT* was noticable. The expression of *NtAnb1* seems to be essential for the accumulation of anthocyanins in tobacco leaf and pedicel. In apple, the efficient induction of anthocyanin biosynthesis by *MdMYB10* is dependent on the coexpression of a bHLH protein [21]. In *MrMYB1-*overexpressing tobacco leaves, both anthocyanin accumulation and the expression of *NtAn1b* are absent, despite the upregulation of late anthocyanin biosynthetic genes [19]. In petunia, *PhAn1*, a bHLH transcription factor, reportedly directly activates the expression of the DFR gene and is specifically regulated by the MYB factor *PhAn2* [56]. In *Arabidopsis*, R2R3-MYB transcription factor *TT2* or *PAP1* regulate the expression of the bHLH reportedly *TT8* [57]. Therefore, in the vegetative organs of tobacco, it can be concluded that exogenous MYB induces the expression of bHLH transcription factor *NtAn1b* and late anthocyanin biosynthetic genes, resulting in the accumulation of anthocyanin.

The mechanisms in reproductive organs are quite different from those in vegetative organs. Downregulated or unchanged expression levels in the three endogenous MYB and bHLH transcription factors were observed in the sepals, petals and ovaries of the transgenic tobacco lines (Fig. 6 C). These results are consistent with those of *MrMYB1-*overexpressing *Arabidopsis*, but inconsistent with *MrMYB1* transgenic tobacco [19]. In *MrMYB1-* overexpressing *Arabidopsis*, the expression of *AtPAP1*, a MYB transcription factor, was suppressed in the roots and leaves but not obviously affected the seeds. However, in tobacco petal, *MrMYB1* overexpression stimulated the expression of endogenous *NtAn2*, *NtAn1a*, and *NtAn1b*. Furthermore, the responses of the structural genes to the overexpression of *LcMYB1* were inconsistent with *MrMYB1-*overexpressing tobacco. Most structural anthocyanin genes were significantly upregulated in the reproductive tissues of the transgenic *MrMYB1* lines in comparison with wild type plants [19]. In the present study, the expression of *NtUFGT* in the transgenic tobacco was upregulated in most of the organs except for petal (Fig. 6 D). Coincident to the much higher expression of *LcMYB1* in leaves of transgenic lines, the upregulation of *NtUFGT* was much obvious in leaf than in pedicel, sepal and ovary. In the petals of transgenic lines, however, only upregulated *NtDFR* expression was observed. These results suggested that the effects of exogenous MYBs appear to differ considerably between species as well as tissues.

## Materials and Methods

### Plant materials and treatments

Twelve different litchi cultivars were used in this study: four non-red skin cultivars [‘Kuixingqingpitian’ (‘KXQPY’), ‘Xinqiumili’ (‘XQML’), ‘Yamulong’ (‘YML’), and ‘Yongxing No. 2’ (‘YX2’)]; two unevenly red cultivars [‘Feizixiao’ (‘FZX’) and ‘Sanyuehong’ (‘SYH’)]; and six evenly red cultivars [‘Meiguili’ (‘MGL’), ‘Baila’ (‘BL’), ‘Baitangying’ (‘BTY’), ‘Guiwei’ (‘GW’), ‘Nuomici’ (‘NMC’) and ‘Guinuo’ (‘GN’)]. These trees were grown in the experimental orchard of South China Agricultural University (Guangzhou, China) received standard horticultural practices, and disease and insect control. Pericarp discs were collected at commercial maturity as reflecting by aril Brix-acid ratio.

Double-layer kraft paper bagging of the clusters on ‘FZX’ commenced at 1 month after full bloom, and the bags were removed at color break. Pericarp samples were taken on the day of bag removal and on the 7 day later. The growth regulators were applied 4 weeks before harvest. Triplicate lots with 3 ‘FZX’ trees were sprayed with abscisic acid (ABA: 25 mg L^−1^), forchlorofenuron (CPPU: 4 mg L^−1^) and tap water (control), respectively. Pericarp discs were sampled when growth regulator spray was applied (day 0) and 1, 3, 7, 14, and 28 days after treatments.

Samples were used to determine anthocyanin content and the transcription levels of the anthocyanin biosynthetic genes and litchi MYBs. Thirty exposed fruit were randomly sampled and the pericarps from 10 individual fruits were pooled into a single replication. Mature leaves, young leaves, young stems, and arils were collected from cultivar ‘NMC’ on July 2, 2011, and pericarps were also collected between May 29 and July 2 at 7-day intervals. All samples were immediately frozen in liquid nitrogen and stored at −80°C until use.

Tobacco (*Nicotiana tabacum*) was used in all genetic transformation. All plants were grown in green houses at 28°C using natural light.

### Anthocyanin analysis

The total anthocyanin content was determined according to the method developed by Fuleki and Francis [58], which involves measuring the absorbance (520 nm) of extracts that have been diluted with pH 1.0 and 4.5 buffers. HPLC analysis of anthocyanins were extracted and determined as previously described [7].

### RNA extraction and cDNA synthesis

Total RNA was extracted from the different tissues of litchi and tobacco using the RNA_OUT_ kit (Tiandz, Beijing, China). DNase I (TaKaRa, Japan) was added to remove genomic DNA, and RNase-free columns (Tiandz,) were used to purify the total RNA. Then, cDNA was synthesized from total RNA (2 μg) using oligo (dT) primers according to the manufacturer's instructions of PrimeScript™ RT-PCR Kit (TaKaRa).

### Cloning of transcription factor genes

Degenerate primers were designed based on the highly conservative amino acid regions of MYB [6]. The cDNAs were synthesized from the total RNA of the mature pericarp of cultivar ‘NMC’ and used as the PCR templates. PCR-amplified products of appropriate length were cloned into T/A cloning vector pMD®20-T (TaKaRa, Japan) and then transformed into *E.coli* DH5α Max Efficiency® Chemically Competent Cells (TaKaRa). Plasmid DNA was isolated from positive *E. coli* cells and digested with *Eco*RI and the final DNA sample was sent to Beijing Genomics Institute for sequencing.

Rapid amplification of cDNA ends (RACE) was performed to obtain the 3′and 5′ ends of the two genes from mature pericarp ‘NMC’ using SMART RACE (Clontech, USA). The primers are shown in Table 2.

**Table 2:** *LcMYB1* 3′ RACE, 5′ RACE, ORF and RT-PCR primers used in this study.

Primer name	Sequence(5′→3′)	function
*LcMYB1*-3′race	TGGCATCAAGTTCCTGTTAGAG	3′RACE
*LcMYB1*-5′race	CTAACAGGAACTTGATGCCATTTTTGTTCGCCAT	5′RACE
*LcMYB1*-F	ATGTCGCATTTACTTGGTG	ORF
*LcMYB1*-R	TTACTTTGCATTGTCTTCTTC	
Q *LcMYB1*-F	GTTGGTCCCTTCAATCTTATC	RT-PCR
Q *LcMYB1*-R	GAAGACGAGGACTCCAACAC	

The promoter region was isolated according to the instructions of the genome walking kit (Takara, Japan). The primer sequences used in the three rounds of amplification are shown in Table 3.

**Table 3:** Primers used to isolated the *LcMYB1* promotor region.

Primer name	Sequence(5′→3′)	Function
MYBSP1-1	GGAAATAGAATCAAATGGATAACGA	First round
MYBSP1-2	CTGCTCTAACAGGAACTTGATGCCA	
MYBSP1-3	TAACAGGAACTTGATGCCATTTTTG	
MYBSP2-1	ACTCCTATGTAACCCTCCGCAGATG	Second round
MYBSP2-2	TCCTATGTAACCCTCCGCAGATGTG	
MYBSP2-3	AAGTGTAGTTTCCACATTCTTTCGT	
MYBSP3-1	TAAGGCAGTTTCACTGTGAGCAGCAC	Third round
MYBSP3-2	TGGACTGTTGAGATGCTATAACCC	
MYBSP3-3	GGTTTACAGGGCTTTGTGCGGAA	

### Sequence analysis

Multiple sequence alignment was performed using ClustalX 1.83 (http://www.ebi.ac.uk) and MEGA5 [59]. *Cis*-acting elements were predicted using the PlantCARE program (http://www.bioinformatics.psb.ugent.be/webtools/plantcare/html/) [60].

### Quantitative real-time PCR analysis

Total RNA from the pericarps of litchi and tobacco was extracted and first strand cDNA was synthesized as described above. The transcription levels of both the litchi and tobacco anthocyanin biosynthetic genes were analyzed using quantitative real-time PCR (qRT-PCR),) with THUNDERBIRD qPCR Mix (TOYOBO, Japan) and ABI 7500 Real-Time PCR Systems (Applied Biosystems, USA) according to the manufacturers' instructions. The primers are shown in Table S1. Each reaction (20 μL final volume,) contained 9.96 μL 2×SYBR® qPCR Mix (TOYOBO), 0.04 μL 50×ROX reference dye, 1.0 μL of each the forward and reverse primers (0.25 μM), 2.0 μL of the cDNA template (corresponding to 50.0 ng of total RNA), and 7.0 μL of RNase-free water. The reaction mixtures were heated to 95°C for 30 s, followed by 40 cycles at 95°C for 10 s, 56°C for 15 s, and 72°C for 35 s. A melting curve was generated for each sample at the end of each run to ensure the purity of the amplified products. The specific qRT-PCR primers were designed using a Primer 5.0 program (PREMIER Biosoft International, Canada) (Table 2). Using these gene-specific primers, each assay amplified a single product of the correct size and demonstrated an acceptable PCR efficiency (approximately 90%). qRT-PCR reactions were normalized to the Ct values for *LcActin* (HQ615689) and *NtACT* (GQ281246) in litchi and tobacco, respectively. The relative expression levels of the target genes were calculated using the formula 2^−ΔΔCT^ [61]. All biological replicates were measured in triplicate.

### Induction of anthocyanins by transient transformation in tobacco

The plasmids used in the transient expression assay were constructed by ligating full-length *LcMYB1* to pEAQ-HT using *Nru* I and *Xho* I. The primers used to amplify the encoding region were: Trans-F: TTCTGCCCAAATTCGCGAATGTCGCATTTACTTGGTGC and Trans-R: AGTTAAAGGCCTCGAGTTACTTTGCATTGTCTTCTTC. The product was recombined with the linearized vector pEAQ-HT (In-Fusion™ Advantage PCR Cloning Kits; Clontech). The constructed vector (pEAQ-LcMYB1) was maintained in *Agrobacterium tumefaciens* strain GV3101. *Agrobacterium* cultures containing pEAQ-LcMYB1 infiltrated into the abaxial leaf surface of *N. tabacum*, as described in Sainsbury et al. [62]. Control infiltrations comprised of pEAQ-HT (empty vector) were pressure infiltrated at the same time. Digital photographs were taken 5 days after infiltration.

### Constructing vector and stable tobacco transformants

To obtain full-length *LcMYB1*, forward (LcMYB1-F: CGGGATCCCGCATGTCGCATTTACTTGGTG) and reverse (LcMYB1-R: CGAGCTCGGTCCATTAAATTACTTTGCATTGTC) primers were designed to amplify the coding regions. Fragments were digested with B*amH*I and S*ac*I and integrated into pBI121 in place of the *gusA* gene sequence. The resulting construct (pBI121- LcMYB1) was introduced into *A. tumefaciens* strain EHA105. The recombinant strains were used to transform *Nicotiana tabacum* K326 using the leaf disk method [63]. Transgenic plants were selected based on kanamycin resistance. Two CaMV 35S:LcMYB1 lines were selected.

## Supporting Information

Figure S1 **Expression of** ***LcMYB2*** **in different tissues (A) and in the pericarp of developmental stages (B) of litchi cultivar ‘NMC’.** *Lcactin* gene was used to normalize expression of the genes under identical conditions. The vertical bars represent standard error of three replicates.
(TIF)

Figure S2 **Images of tobacco lines containing** ***LcMYB1*** **allele with CaMV 35S promoter.** A-E: pigment formation during transformed process; F and G: pigmented leaves during seedling domestication.
(TIF)

Table S1 **Primers for real-time PCR analysis.**
(DOC)
