# Supplementary figures and images for: LcMYB1 Is a Key Determinant of Differential Anthocyanin Accumulation among Genotypes, Tissues, Developmental Phases and ABA and Light Stimuli in Litchi chinensis
Source: PLoS One. 2014 Jan 21;9(1):e86293. doi: 10.1371/journal.pone.0086293 (PMC3897698; doi:10.1371/journal.pone.0086293)

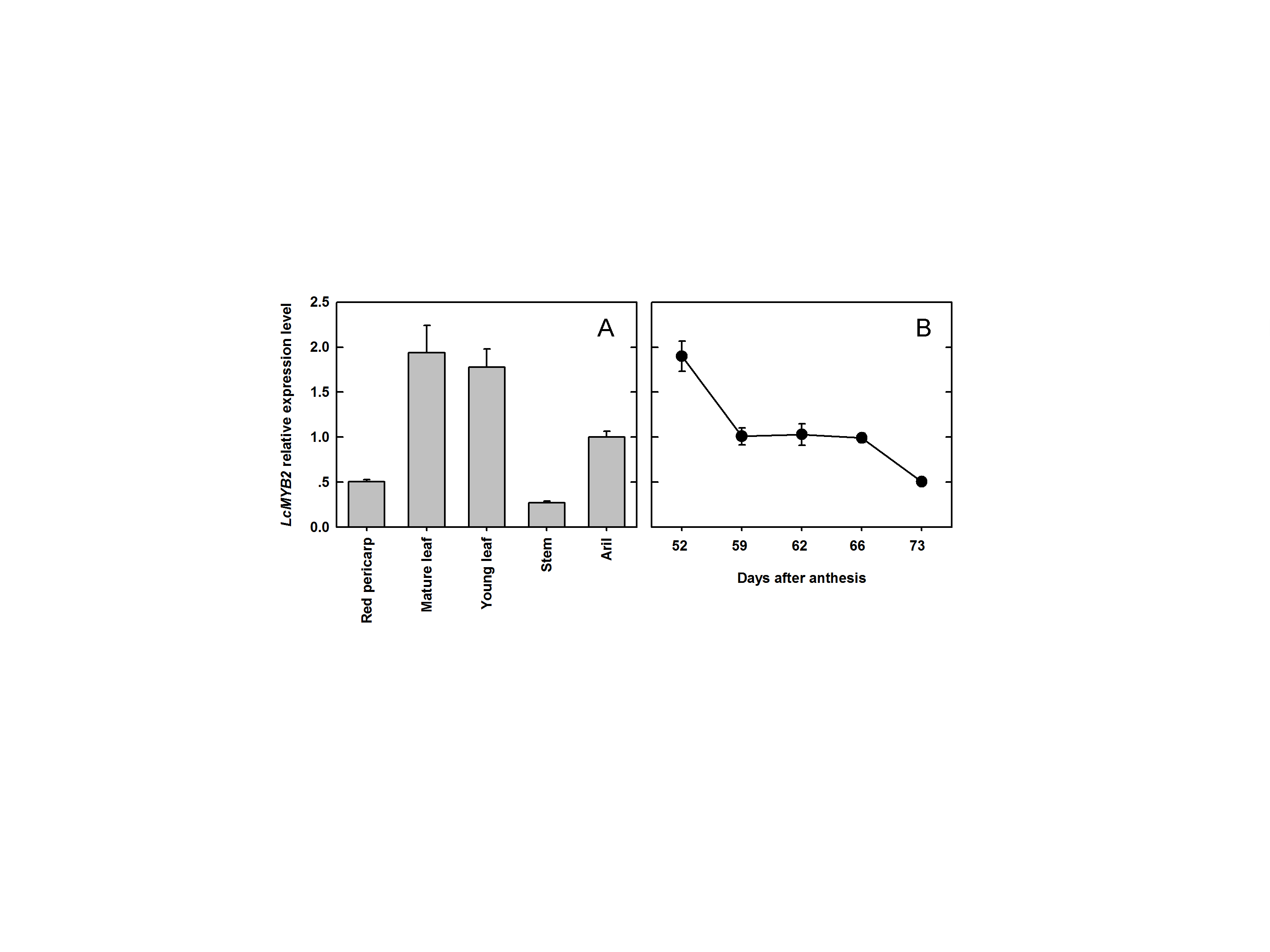

Supplement: Figure S1 — Expression of LcMYB2 in different tissues (A) and in the pericarp of developmental stages (B) of litchi cultivar ‘NMC’. Lcactin gene was used to normalize expression of the genes under identical conditions. The vertical bars represent standard error of three replicates. (TIF) [file pone.0086293.s001.tif]

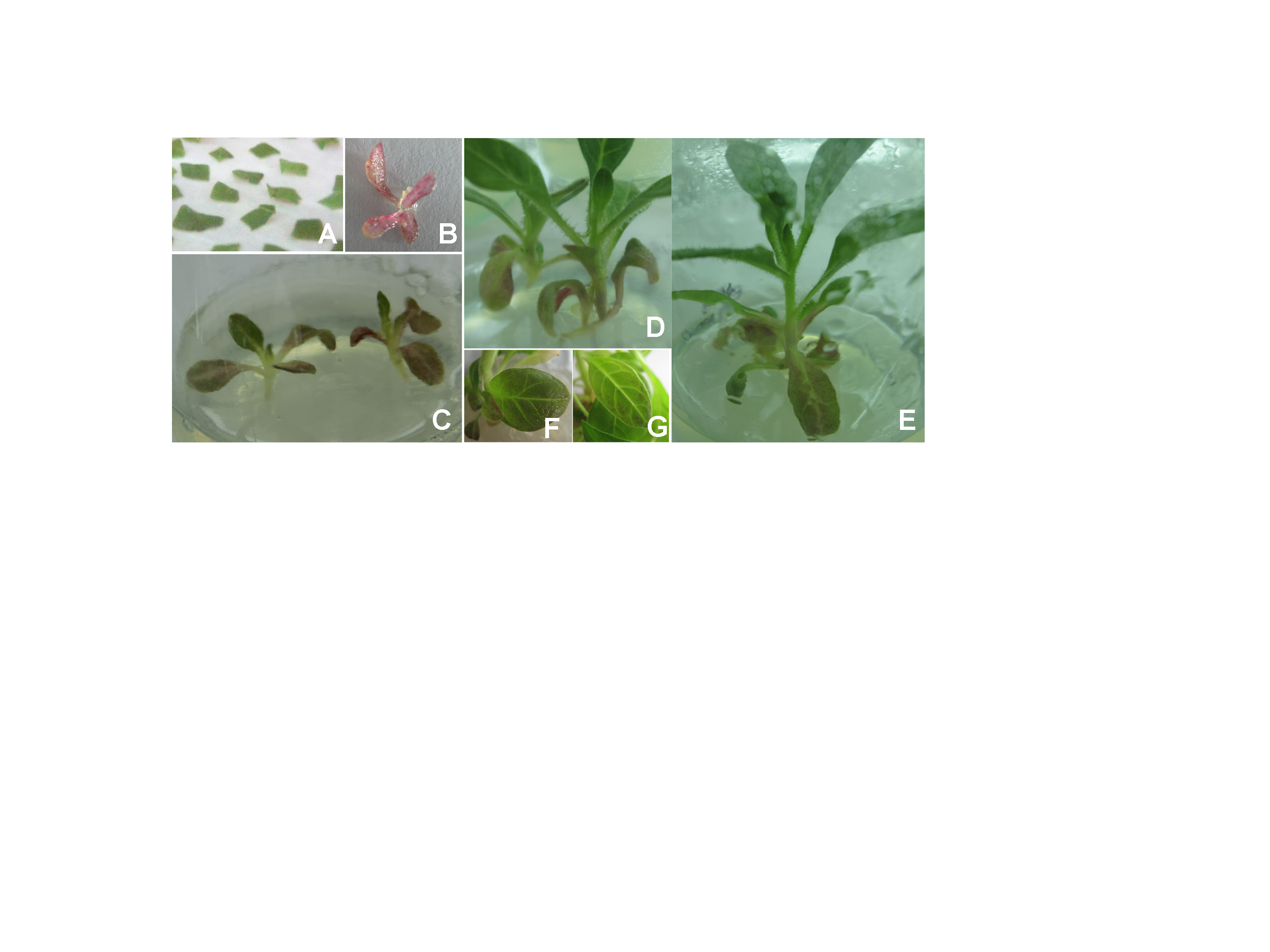

Supplement: Figure S2 — Images of tobacco lines containing LcMYB1 allele with CaMV 35S promoter. A-E: pigment formation during transformed process; F and G: pigmented leaves during seedling domestication. (TIF) [file pone.0086293.s002.tif]
