# Supplementary material for: LcMYB1 Is a Key Determinant of Differential Anthocyanin Accumulation among Genotypes, Tissues, Developmental Phases and ABA and Light Stimuli in Litchi chinensis
Source: PLoS One. 2014 Jan 21;9(1):e86293. doi: 10.1371/journal.pone.0086293 (PMC3897698; doi:10.1371/journal.pone.0086293)
Supplement: Table S1 — Primers for real-time PCR analysis. (DOC) [file pone.0086293.s003.doc]

Table S1 Primers for real-time PCR analysis

| Genes | GenBank ID | Forward primer (5' to 3') | Reverse primer (5' to 3') |
| --- | --- | --- | --- |
| *NtAn1a* | HQ589208 | ACCATTCTCGAACACCGAAG | TGCTAGGGCACAATGTGAAG |
| *NtAn1b* | HQ589209 | CTTGAACACTTCTCAAACCGA | TGCTAGGGCACAATGTGAAG |
| *NtUFGT* | FG627024 | GAGTGCATTGGATGCCTTTT | CCAGCTCCATTAGGTCCTTG |
| *NtAn2* | FJ472647 | GAAGAAAGGTGCATGGACTG | TCTGCAGCTCTTTCTGCATC |
| *NtPAL* | X78269 | ATTGAGGTCATCCGTTCTGC | TCTGCAGCTCTTTCTGCATC |
| *Nt4CL* | U50845 | TCATTGACGAGGATGACGAG | TGGGATGGTTGAGAAGAAGG |
| *NtCHS* | AF311783 | TTGTTCGAGCTTGTCTCTGC | AGCCCAGGAACATCTTTGAG |
| *NtCHI* | AB213651 | GTCAGGCCATTGAAAAGCTC | CTAATCGTCAATGCCCCAAC |
| *NtF3H* | AB289450 | CAAGGCATGTGTGGATATGG | TGTGTCGTTTCAGTCCAAGG |
| *NtDFR* | EF421429 | AACCAACAGTCAGGGGAATG | TTGGACATCGACAGTTCCAG |
| *NtANS* | AB289447 | TGGCGTTGAAGCTCATACTG | GGAATTAGGCACACACTTTGC |
| *NtACT* | GQ281246 | AATGGAACTGGAATGGTCAAGGC | TGCCAGATCTTCTCCATGTCATCCCA |
| *LcCHS* | [GU288820.1](http://www.ncbi.nlm.nih.gov/nucleotide/283827863?report=genbank&log$=nucltop&blast_rank=2&RID=9YFPHG4M016) | GACATTGTGGTGGTGGAGGT | TATTTAGCGAGACGGAGGAC |
| *LcCHI* | HQ402910 | CGGAGTTTACTTGGAGGATGT | CAGTGACCTTCTCAGAGTATTG |
| *LcF3H* | HQ402911 | GGTGGATAGATGTGACAAAGGAGT | GGTTGTGGGCATTTTGGATAGTA |
| *LcDFR* | HQ402912 | ATAAAGCCAACTATCAATGGGAT | AGCCCATATCACTCCAGCAAGT |
| *LcANS* | HQ402913 | AGGAAGTTGGTGGTCTGGAAG | CCGTTGCTGAGGATTTCAATGGTG |
| *LcUFGT* | HQ402914 | GCCACCAGCGGTTCCTAATA | ATGCCTCTGCTACTGCTACAATCT |
| *Lcactin* | HQ615689 | ACCGTATGAGCAAGGAAATCACTG | TCGTCGTACTCACCCTTTGAAATC |
